# Primary Angiitis of the Central Nervous System: From Psychiatry to Neurology

**DOI:** 10.1155/2019/8074258

**Published:** 2019-10-31

**Authors:** Joel Leonardi, Christian Saleh, Phillip Jaszczuk, Kristine A. Blackham, Sabrina Zechel, Stefanie Wilmes, Margret Hund-Georgiadis

**Affiliations:** ^1^REHAB Basel, Clinic for Neurorehabilitation and Paraplegiology, Basel, Switzerland; ^2^Department of Radiology, Division of Diagnostic and Interventional Neuroradiology, University Hospital Basel, Basel, Switzerland; ^3^Institute of Neuropathology, University Medical Center Göttingen, Göttingen, Germany

## Abstract

We present a case of a 54-year-old man with primary angiitis of the central nervous system (PACNS) who was initially admitted to a psychiatric clinic with a diagnosis of delirium. We discuss the difficulty in establishing the diagnosis of PACNS and provide the reader with some recommendations on how to promptly and correctly diagnose this disease in order to avoid potentially lethal outcomes.

## 1. Introduction

Primary angiitis of the central nervous system (PACNS) is a vasculitis of unknown origin confined to the brain and spinal cord [1]. PACNS was first described in the late 1950s by Cravioto and Feigin [2]. Histopathology is characterized by segmental granulomatous inflammation of small- and medium-sized arteries and veins of the CNS. Definitive diagnosis is made by biopsy.

All the vasculitides that affect the central nervous system (CNS) are characterized by inflammation and necrosis of blood vessels and may be primary or secondary. They may cause tissue ischemia, which can have a variety of clinical presentations. Isolated CNS vasculitis is a known, rare disease entity with a variety of presentations; headache, dementia, lethargy, seizures, or multifocal neurological deficits [3]. Incidence rate of 2.4 person‐years was reported by Salvarani et al. [4].

The clinical presentation is highly unspecific ranging from psychiatric to neurological symptoms, therefore, frequently delaying a prompt diagnosis and treatment with potentially lethal consequences. We describe a patient initially admitted to a psychiatric hospital, but diagnosed subsequently with PACNS. In addition, we provide a brief review of the literature.

## 2. Case Presentation

**Figure 1:**
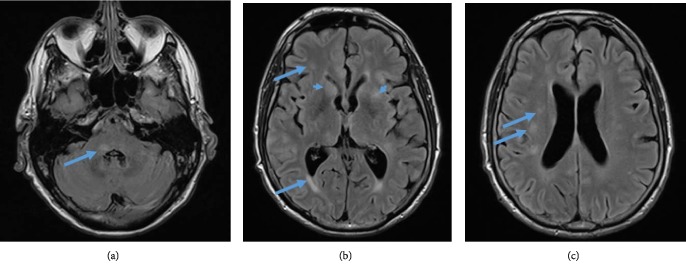
Initial MRI brain, FLAIR transversal images with multifocal abnormal hyperintensities (arrows). (a) Middle cerebellar peduncle. (b) Periventricular and subcortical white matter, right caudate nucleus, and left lentiform nucleus. (c) Deep white matter.

**Figure 2:**
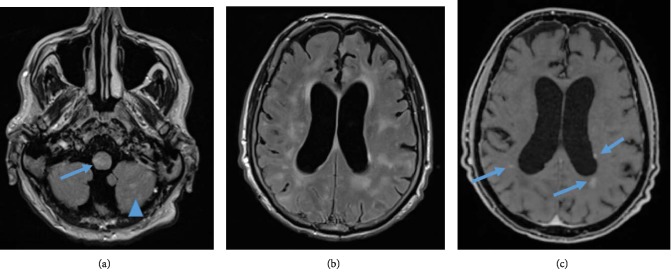
MRI brain 4 months after presentation. FLAIR transversal images (a and b) and post contrast image (c) demonstrating marked brain atrophy and new abnormal hyperintensities in the spinal cord (arrow, a) and cerebellum (arrowhead, a) , throughout the periventricular and subcortical white matter (b) and new enhancement (arrows, c).

**Figure 3:**
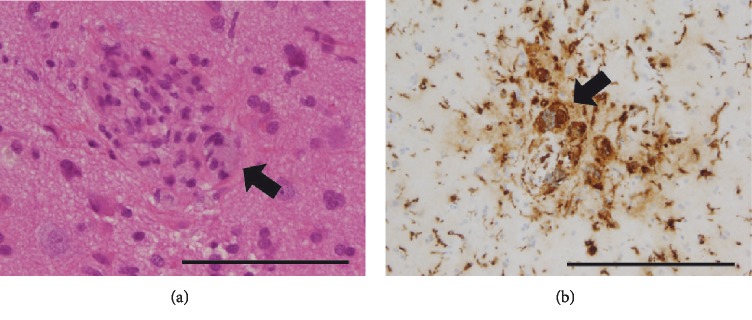
(a) HE staining of brain biopsy showing a nearly obliterated vessel with perivascular lymphocytes and a giant cell (arrow points ta the giant cell). Scale bar: 100 *µ*m. (b) Immunostaining against Kim1P positive monocytes and monocytic giant cells (arrow points at giant cells, counterstaining of nuclei with hemalum). Scale bar: 100 *µ*m

A 54-year-old Caucasian man presented with agitation and confusion. Glasgow coma score (GCS) was 14 out of 15. The patient was reported to be in his baseline state (of generally good health) when his family last saw him one week prior. The initial working diagnosis was delirium. The clinical picture worsened rapidly when the patient developed hypertension, tremor, meningism, and an increase in C-reactive protein (CRP 143 mg/l, reference: 0–1), without fever. MRI FLAIR—sequences presented abnormal but nonspecific bilateral hyper‐intensities supra‐ and infratentorially in the gray and white matter (periventricular and subcortical white matter), the basal ganglia, the thalamus, the middle cerebellar peduncle, and in the brainstem (Figures 1(a)–1(c)). A lumbar puncture showed a leukocytosis 9 × 10−/l (reference: 0–5), a total protein 789 mg/l (reference: 200–400), a lactate level of 3.2 mmol/l (reference: 1.2–2.1). Intravenous antibiotic (ceftriaxone 2 g/day) and antiviral (acyclovir 750 mg/day) treatment was started, but once infection‐screening and biochemistry results showed no evidence of infection it was stopped. Over the following weeks, the patient developed a progressive tetraparesis, rigidity, and mutism, with a GCS fluctuating between 6 and 10. Repeated autoimmune, vasculitis, viral, prion, and bacterial screenings were negative. Digital subtraction angiography (DSA) showed no specific evidence of vasoconstriction or vasculitis. A follow up brain MRI showed progressive patchy and confluent T2w signal hyperintensities, again supra‐ and infratentorially and, again, of the gray and white matter. There were new lesions in the upper cervical spinal cord and new small areas of parenchymal enhancement as well as marked brain atrophy, indicating a progressive and prolonged disease process (Figures 2(a)–2(c)). In the absence of infection and even in the absence of vasculopathy on DSA, these findings were consistent with vasculitis. Brain biopsy (Figures 3(a) and 3(b)) from the right frontal lobe revealed perivascular and transmural leukocyte infiltration of small vessels composed of CD8 positive cytotoxic T cells, macrophages, and giant Langerhans type cells. CD20 positive B cells were almost absent. Moreover, reactive astrogliosis as well as microglial activation were found. There was no vessel wall fibrinoid necrosis. A biopsy of the dura mater showed no abnormalities. A diagnosis of suspected primary central nervous system angiitis was made. The etiology of inflammation could not be precisely determined despite vast immunohistological screening. Treatment with methylprednisone 1 g/d i.v., intravenous immunoglobulin (IVIG) 27.5 g/daily over 4 days and i.v. cyclophosphamide (three cycles of 15 mg/kg) was started. The patient recovered gradually with slight improvement of neurological deficits.

## 3. Discussion

Despite a large volume of literature on PACNS definitive diagnosis is frequently delayed resulting in potentially lethal consequences. One major reason is the unspecific clinical presentation ranging from recurrent headaches, altered consciousness, seizures, stroke, and a lack of high clinical suspicion ([Supplementary-material supplementary-material-1]). Imaging findings of PACNS are variable and nonspecific [14]. Although MRI examinations are sensitive (abnormal in 90% to 100% of patients), the typical findings are nonspecific. The lesions seen include discrete or diffuse supra‐ and infratentorial lesions most commonly involving the superficial white matter, followed by the deep gray matter, the deep white matter, and the cerebral cortex, in decreasing order of frequency [5]. Infarctions may be seen in approximately 50% of cases and mass lesions in as many as 15% of cases. These imaging characteristics can be mistaken for malignancy, multiple sclerosis, small vessel ischemic disease, and atherosclerosis among others. Gadolinium enhancement may occur in as many as one‐third of cases [5]. The overall sensitivity of angiography in detecting PACNS has been estimated to be between 50% and 90%. Angiograms have a limited specificity as there is a range of noninflammatory vasculopathies, which can cause angiographic findings similar to those seen with PACNS [6]. In a recent systematic review of 127 articles including 701 patients, only 248 patients had both angiography and biopsy performed. Of those, only 32 patients (4.6%) had the classic angiographic appearance of vasculitis with concurrent histopathological vasculitis [7].

## 4. Conclusion

A high degree of clinical suspicion is needed for a prompt diagnosis of PACNS in patients manifesting with (transient) multi‐focal symptoms associated with encephalopathy [13]. Vasculitis screening, imaging (MRA, DSA) to consider prompt initiation of immune‐modulatory treatment is mandatory.

## Additional Points

- PACNS is to be suspected with an array of symptoms ranging from psychiatric to neurologic manifestations (especially when associated with encephalopathy) that cannot be clearly localized.
- Prompt diagnostic work up including vasculitis screening and MR‐Angiography is recommended by suspicion of PACNS.
- Brain biopsy is considered the gold standard investigation in establishing the diagnosis of PACNS; however, it will confirm the diagnosis only in circa 60% of patients [13].
- PACNS should be treated as a diagnosis of exclusion using a broad differential diagnosis. When there is a high degree of certainty of PACNS, immune‐modulatory treatment should be started promptly.

## Supplementary Materials

Supplementary Materials Supplementary Table S1: literature findings.
