# Supplementary material for: Primary Angiitis of the Central Nervous System: From Psychiatry to Neurology
Source: Case Rep Neurol Med. 2019 Oct 31;2019:8074258. doi: 10.1155/2019/8074258 (PMC6875412; doi:10.1155/2019/8074258)
Supplement: Supplementary Materials — Supplementary Table S1: literature findings. [file 8074258.f1.pdf]

**Author (Reference)**	**Gender/age**	**Clinic**	**Imaging/Findings**	**Biopsy/ Results**	**Treatment**
8	M/28 yrs	Headache<br>Seizure<br>Hemiplegia	CA/vasculitis typical changes	No	Steroid
	F/16 yrs	Fever<br>Dysphagia<br>Seizure	MRI/Thalamus infarct	No	Steroid
	F/38 yrs	Dysarthria<br>Ataxia<br>Quadriparesis	CA/negativ	Yes/ confirming vasculitis	Steroid
9	F/20 yrs	Headache<br>Vomiting<br>Diplopia<br>Upper Motor Symptoms	MRI/Supra-intratentorial lesions	Yes/ confirming vasculitis	Steroid<br>Cyclophosphamide<br>Azathioprin
10	M/54 yrs	Hemiparesis<br>Headache<br>Aphasia	MRI-A/vasculitis typical changes	Yes/ confirming vasculitis	Steroid<br>Cyclophosphamide
	W/55 yrs	Hemiparesis<br>Aphasia<br>Stupor	CA/Vasculitis appearing changes	Yes/ confirming vasculitis	Steroid
	M/35 yrs	Hemiparesis<br>Language disorder	MRI/hyperintense lesions in frontal lobe, paraventricular area	Yes/ confirming vasculitis	Steroid<br>Cyclophosphamide
11	M/42 yrs	Stereotypic tingling spells in right hand	Gradient echo MRI/multiple petechial hemorrhages	Yes/ confirming vasculitis	Steroid<br>Cyclophosphamide
12	F/ 70 yrs	Confusion<br>Disorientation<br>Memory loss	CT/areas of cerebral edema	Autopsy confirmed vasculitis	Steroid

Table 1: Literature findings

Legend: M: Male; F: Female; CA: Cerebral angiography; MRI-A: MRI-Angiography; yrs: years
